# Subjective and Objective Outcomes in Patients With COPD After Pulmonary Rehabilitation – The Impact of Comorbidities

**DOI:** 10.3389/fphys.2019.00286

**Published:** 2019-03-22

**Authors:** Maria Charikiopoulou, Pantelis Theodoros Nikolaidis, Beat Knechtle, Thomas Rosemann, Aggeliki Rapti, Georgia Trakada

**Affiliations:** ^1^Pulmonary Rehabilitation Department, General Hospital for Chest Diseases of Athens “SOTIRIA”, Athens, Greece; ^2^2nd Pulmonary Department, General Hospital for Chest Diseases of Athens “SOTIRIA”, Athens, Greece; ^3^Exercise Physiology Laboratory, Nikaia, Greece; ^4^Institute of Primary Care, University of Zurich, Zurich, Switzerland; ^5^Division of Pulmonology, Department of Clinical Therapeutics, School of Medicine, Alexandra Hospital, National and Kapodistrian University of Athens, Athens, Greece

**Keywords:** dyspnea, quality of life, six minute walking test, cardiopulmonary exercise test, lung disease

## Abstract

**Background:** Chronic obstructive pulmonary disease (COPD) is a heterogeneous disease with multiple systemic manifestations and comorbidities, which contribute independently to its total morbidity and mortality. Pulmonary rehabilitation is an evidence-based intervention that is indicated for COPD patients who remain symptomatic, despite optimal pharmacological therapy. Although it is well documented in pure COPD, the role of pulmonary rehabilitation is uncertain in coexisting comorbidities. The aim of the present study was to clarify the effect of a pulmonary rehabilitation program in COPD patients with concomitant comorbidities.

**Methods:** Thirty two patients with COPD were evaluated before and after a comprehensive pulmonary rehabilitation program, in terms of dyspnea, quality of life (QOL), pulmonary function tests and exercise capacity. The patients were also divided into two groups, according to the presence or the absence of comorbidities. Patients with none or only one comorbidity (Group 1, *n* = 11) were compared to those who had two or more comorbidities (Group 2, *n* = 21).

**Results:** All patients significantly improved in dyspnea, as expressed by modified Medical Research Council scale and the COPD assessment Test (*p* < 0.001), QOL as assessed by the St. George respiratory questionnaire (*p* < 0.001) and exercise tolerance in six minute walking test (*p* < 0.001). Peak oxygen uptake relatively increased and body mass decreased in Group 1 compared to Group 2 (*p* < 0.05).

**Conclusion:** Pulmonary rehabilitation in COPD seems to be beneficial for all patients, independently of the presence, the number or the nature of their comorbidities. Thus, the presence of comorbidities must not represent an exclusion criterion for patients that are referred to pulmonary rehabilitation programs.

**Trial Registration:** Current controlled trials ISRCTN14648515 Retrospectively registered 15 February 2018.

## Introduction

Chronic obstructive pulmonary disease (COPD) is a progressive disease associated with significant morbidity and mortality. The latest Global Initiative for Obstructive Lung Disease (GOLD) defines the disease as: “A common preventable and treatable disease, characterized by persistent airflow limitation that is usually progressive and associated with an enhanced chronic inflammatory response in the airways and the lung to noxious particles or gases. Exacerbations and comorbidities contribute to the overall severity in individual patients” (Global strategy for the diagnosis, management and prevention of COPD, global initiative for chronic obstructive lung disease (GOLD), 2016).

Several studies demonstrate that 86–98% of COPD patients have at least one comorbidity, with the average number per individual varying between from 1.2 to 4.0 (Putcha et al., 2013, 2015). Comorbidities often contribute to dyspnea, limitation of exercise capacity, deterioration of quality of life (QOL), increased healthcare utilization and cost, and increased exacerbation and mortality risk (Patel and Hurst, 2011). COPD patients are more likely to die from a comorbid disease than COPD itself, as “only 40% of the deaths are definitely or probably related to COPD, with the remainder being mostly unrelated to COPD (50%) or unknown (9%)” (McGarvey et al., 2007). Based on this evidence, current guidelines for the management of COPD, besides treating chronic airflow limitation with traditional pharmacological therapy, adopt a more holistic approach by incorporating the assessment and appropriate treatment of comorbid diseases in order to ameliorate the health status and the prognosis of the patients (GOLD Global strategy for the diagnosis, management and prevention of COPD, global initiative for chronic obstructive lung disease (GOLD), 2016).

Common comorbidities in patients with COPD include cardiovascular disease (CVD) (such as arterial hypertension, systemic venous thromboembolism (VTE), stroke, heart failure, coronary heart disease, arrhythmias and pulmonary hypertension), lung cancer and other cancers, psychiatric diseases (such as anxiety and depression disorders), metabolic conditions (such as malnutrition, obesity, diabetes mellitus, dyslipidemia), anemia, osteoporosis, musculoskeletal dysfunction, sleep disorders and gastroesophageal reflux (Patel and Hurst, 2011). Some of these comorbidities (pulmonary artery disease and malnutrition) are directly caused by COPD (secondary to COPD), whereas others share common risk factors, such as lifestyle factors (e.g., smoking), environmental exposures (place of work, air pollution etc.), and genetic susceptibility, and are associated with similar pathophysiological mechanisms, like airway and systemic inflammation, lung hyperinflation and endothelial dysfunction and oxidative stress (Patel and Hurst, 2011).

Pulmonary rehabilitation is an evidence-based intervention that is indicated for COPD patients who remain symptomatic – despite optimal pharmacological therapy – to reduce dyspnea, increase exercise capacity and improve QOL (Spruit et al., 2013). Exercise training is the best available way of improving muscle function and exercise tolerance in patients with COPD (Wu et al., 2017). Moreover, regular physical activity is recommended and is beneficial for patients with CVD, musculoskeletal disease, obesity, diabetes mellitus and most other chronic medical conditions (Ciolac, 2013). However, a review on this topic concluded inconsistent data about pulmonary rehabilitation outcomes in COPD patients with comorbidities (Franssen and Rochester, 2014). For instance, patients with multiple comorbidities would present limited benefits from their participation in pulmonary rehabilitation (Crisafulli et al., 2008), whereas most comorbidities were not related to rehabilitation outcomes (Crisafulli et al., 2010). In most studies reported in the abovementioned review, outcomes were evaluated with functional exercise capacity as measured by six minute walking test (6MWT) and not with peak oxygen uptake (VO_2_peak) as measured during incremental exercise. Some studies proved worse outcomes in patients with cardiovascular, metabolic or psychological comorbid conditions, whereas others did not confirm this or reported opposite effects (Franssen and Rochester, 2014). Such knowledge would be of great practical value for practitioners working with COPD as it would allow them prescribing optimal pulmonary rehabilitation programs.

Therefore, the aim of our study was to further clarify pulmonary rehabilitation outcomes in stable COPD patients with coexisting comorbidities, in terms of specific diseases and total number. The principal end points were QOL, degree of dyspnea and aerobic capacity. We assessed exercise capacity using both 6MWT and VO_2_peak, in order to evaluate possible differences in results between the two methods.

## Materials and Methods

**FIGURE 1:**
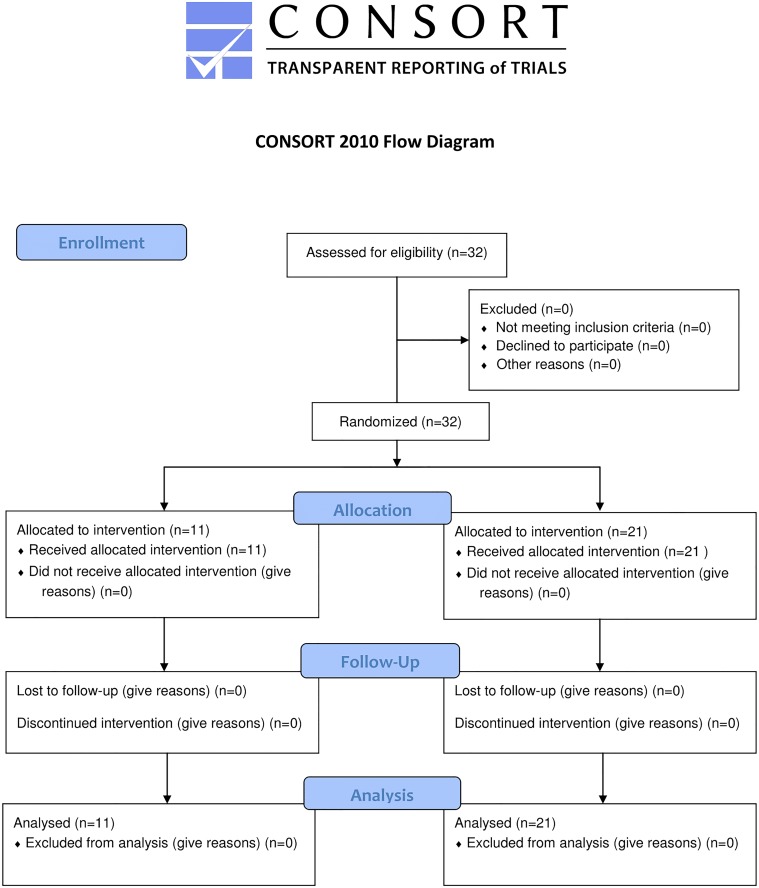
CONSORT flow graph showing the enrollment, allocation, follow-up and analysis of participants.

Thirty two patients with COPD (25 men and 7 women, age 66 ± 6 years, body mass index (BMI) 27.14 ± 4.88 kg/m^2^) (Table 1), sample of convenience, were selected for the present study from our outpatient clinics in the Division of Pulmonology, to the Department of Clinical Therapeutics of the National and Kapodistrian University of Athens School of Medicine, at Alexandra Hospital of Athens. COPD was diagnosed by history, physical examination, and standard pulmonary function tests according to GOLD criteria (Global strategy for the diagnosis, management and prevention of COPD, global initiative for chronic obstructive lung disease (GOLD), 2016). Also, at baseline each patient underwent detailed general medical history – including the number, the type and the medications of other coexisting diseases. Finally, each patient signed a written informed consent and the study protocol was approved by the Ethical Committee of our hospital. The study adheres to the CONSORT criteria (Figure 1). All data are available in the Supplementary Material.

**Table 1:** Baseline characteristics of participants.

Parameters	Total (*n* = 32)	Group 1 (*n* = 11)	Group 2 (*n* = 21)
Patients (number (%), males/females)	25 (78%)/7 (22%)	10 (91%)/1 (9%)	15 (71%)/6 (29%)
Age (years)	66.03 ± 6.03	64.36 ± 5.45	66.91 ± 6.26
BMI (kg/m^2^)	27.14 ± 4.88	25.36 ± 4.73	28.07 ± 4.81
FEV_1_ (L)	1.297 ± 0.605	1.09 ± 0.53	1.40 ± 0.63
FEV_1_ (% predicted)	43.08 ± 15.07	35.78 ± 14.12	48.02 ± 15.16^∗^
FEV_1_/FVC (%)	51.67 ± 10.25	45.43 ± 6.93	54.94 ± 10.30^∗^
RV/TLC (%)	54.87 ± 19.68	68.20 ± 28.98	47.76 ± 5.73^∗^
TLCO (% predicted)	38.2 ± 22.8	21.7 ± 18.0	61.8 ± 20.0^∗^
MRC	2.5 ± 0.9	2.5 ± 1.0	2.5 ± 0.9
CAT	16.9 ± 9.3	15.6 ± 5.7	17.5 ± 10.8
SGRQ	47.3 ± 19.0	41.1 ± 16.2	50.5 ± 20.0
GOLD Severity (number, A/B/C/D)	1/9/14/8	0/2/4/5	1/7/10/3
6MWT (m)	280 ± 81	297 ± 95	271 ± 74
Workload (W)	58.4 ± 31.5	56.1 ± 29.6	59.5 ± 33.1
VO_2_peak (mL min^-1^ kg^-1^)	13.2 ± 4.1	16.9 ± 5.0	11.9 ± 3.7^∗^


*BMI: Body Mass Index, FEV_1_: Forced Expiratory Volume in 1 s, FVC: Forced Vital Capacity, RV: Residual Volume, TLC: Total Lung Capacity, TLCO: Diffusing Capacity, MRC: modified Medical Research Council scale, CAT: COPD Assessment Test, SGRQ: St. George Respiratory Questionnaire, GOLD: Global Strategy for the diagnosis, management, and prevention of COPD, 6MWT: 6 min Walking Test, VO_*2*_peak: Peak Oxygen Uptake. ^∗^*p* < 0.05*.

Eligible participants were 25 men and 7 women, with mean age 66 ± 6 years-old (range: 49–76) who met the following inclusion criteria: (1) COPD diagnosis of any stage (A, B, C, D), (2) stable condition, (3) optimal bronchodilation therapy, (4) optimal therapy of comorbidities, (5) reduced exercise capacity, and (6) completed successfully all parts of experimental intervention. Stable COPD was defined as no history of exacerbation in last 2 months prior to the study (Biswas et al., 2017).

The patient population was divided in two groups, according to the presence or the absence of comorbidities. The comorbidities were both self-reported and confirmed in the medical records of the patients. Patients with none or only one comorbidity (*n* = 11, Group 1) were compared to those who had two or more comorbidities (*n* = 21, Group 2), in terms of dyspnea, QOL and exercise capacity, before and after a pulmonary rehabilitation program. The characteristics of each group were presented in Table 1. The experimental intervention was administered twice a week, for 13 weeks between 1 November 2012 and 31 December 2014 at the Division of Pulmonology, Department of Clinical Therapeutics of the National and Kapodistrian University of Athens School of Medicine, at Alexandra Hospital of Athens. Comorbidities were assessed using patient-report of a physician diagnosis.

At baseline, each patient underwent clinical examination, arterial blood gases analysis (GEM Premier 3500, United States), and Pulmonary Function Tests (PFTs) (Master screen Diffusion, Jaeger, Germany): spirometry (pre/post-bronchodilation), diffusing capacity and static lung volumes. Dyspnea was assessed by both the modified Medical Research Council (MRC) scale (Simsic et al., 2018) and the COPD Assessment Test (CAT) (Jo et al., 2018). QOL was assessed by the St. George Respiratory Questionnaire (SGRQ) (Oliveira and Marques, 2017). BMI was calculated using patient’s weight in kilograms (kg) divided by his or her height in meters squared.

Six minute walking test (Reychler et al., 2016) and standard cardiopulmonary exercise test (Ergoline Variable 500, Viasys Health Care, Jaeger, Germany) were also carried out, while breathing room air. Expiratory oxygen and carbon dioxide fractions, work rate, airflow, cardiac frequency and oxygen saturation were continuously recorded. The results were obtained (1) at rest, (2) after 1 min of unloaded exercise, and (3) during incremental exercise. The incrementation rate was 25 W per minute. The pedaling frequency was constant at 60 revolutions per min. Each patient exercised against progressive workloads up to symptom-limited, maximum exercise capacity. All indications to stop the test were known by the personnel involved in the testing. The procedure was performed under the supervision of a physician trained in conducting exercise tests and in advanced cardiopulmonary resuscitation (CPR). Full CPR equipment was available in the laboratory.

After the initial assessment, each patient started an individualized rehabilitation program, at the Pulmonary Rehabilitation Department of Sotiria Hospital, twice a week, for a period of 13 weeks (Table 2). The program included (1) interval, endurance exercise training using cycle ergometer to 100% of WRmax of each patient, switching between exercise and rest, for 40 min, (2) continuous endurance exercise training using treadmill for 10–20 min, (3) lower and upper extremity strengthening exercise training-conditioning using mainly weight lifting for 5–15 sets, (4) breathing retraining (pursed lips breathing technique) and (5) respiratory muscle training. Education, diet and psychological support were provided by specialized personnel during the experimental period. Other important therapeutic modalities that were stressed during the program included smoking cessation, oxygen therapy, and use of bronchodilators and antibiotics. At the end of the 13 weeks period of training, the patients were reassessed using the same testing protocol.

**Table 2:** Characteristics of pulmonary rehabilitation program.

General approach	The program was multimodal and evolutionary for each participant.
Overall sessions	Twenty-five sessions for each participant.
Content	Each session consisted by both physical therapy modalities for the respiratory system and exercise.
	Exercise included both aerobic (treadmill, cycle ergometer and arm crank ergometer) and resistance training (3 set × 10–15 repetitions for 6–8 exercises of main muscle groups). Oxygen supply was provided continuously to maximize effort and exercise intensity maintaining hemoglobin saturation higher than 90%.
	Physical therapy included relaxation techniques for dyspnea, pursed lips breathing technique, exercise of diaphragm/respiratory muscles and effective cough.
Personnel	Participants were instructed by physical therapists under the guidance of physician and nurse.
Frequency	Two sessions weekly (Monday and Thursday morning). The participants were encouraged and guided to perform an additional session by their own.
Exercise intensity	Dyspnea was continuously monitored during each session using Borg scale and was considered to regulate exercise intensity.


The values of all functional parameters were expressed as mean and standard deviation (M ± SD). An independent *t*-test examined differences between the two experimental groups. A between-within subjects analysis of variance examined the main effect of time and the time × group interaction on QOL, dyspnea and exercise tolerance. The magnitude of the differences between experimental groups was examined using effect size eta square (η^2^) and was evaluated as following: small (0.010 < η^2^≤ 0.059), moderate (0.059 < η^2^≤ 0.138) and large (η^2^ > 0.138) (Cohen, 1988). The correlations among the pre-post changes in exercise tolerance, dyspnea or QOL, and the baseline COPD stage were examined using the Spearman correlation coefficient *rho*. The acceptable type I error was set at *p* < 0.05.

## Results

The presence of comorbidities was very common (*n* = 28, 87.5% of patients) and included cardiovascular, diabetes mellitus, osteoporosis, psychological, and sleep disorders and cancers (Table 3). The two more frequently observed comorbidities were CVD and diabetes mellitus (*n* = 22, 68.8% and *n* = 17, 53.1%, respectively).

**Table 3:** Prevalence of selected comorbidities in participants.

Comorbidity	Frequency (*n*)	Percentage (%)
Cardiovascular	22	68.8
Diabetes mellitus	17	53.1
Sleep apnoea and other lung diseases	5	15.6
Osteoporosis	4	12.5
Lung cancer	1	3.1
Other cancers	5	15.6
Psychiatric	3	9.4
Chronic kidney failure	1	3.1
Gastrointestinal reflux	0	0


**FIGURE 2:**
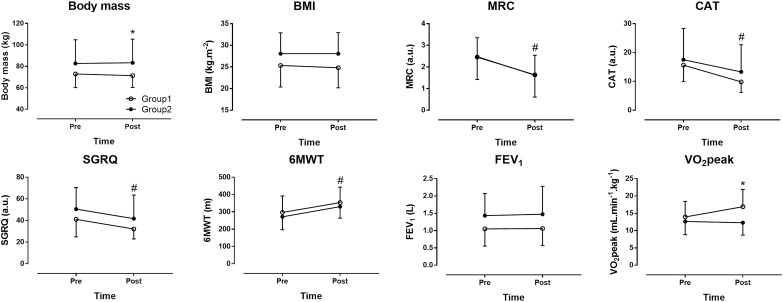
The effect of pulmonary rehabilitation on dyspnea, quality of life (QOL) and aerobic capacity. Group 1 = patients with none or only one comorbidity, Group 2 = patients with two or more comorbidities, BMI = body mass index, FEV_1_ = forced expiratory volume in 1 s, MRC = modified Medical Research Council scale, CAT = COPD assessment test, SGRQ = St. George respiratory questionnaire, GOLD = global strategy for the diagnosis, management, and prevention of COPD, 6MWT = 6 min walking test, VO_2_peak = peak oxygen uptake. ^∗^group × time interaction at *p* < 0.05, #main effect of time at *p* < 0.05.

**FIGURE 3:**
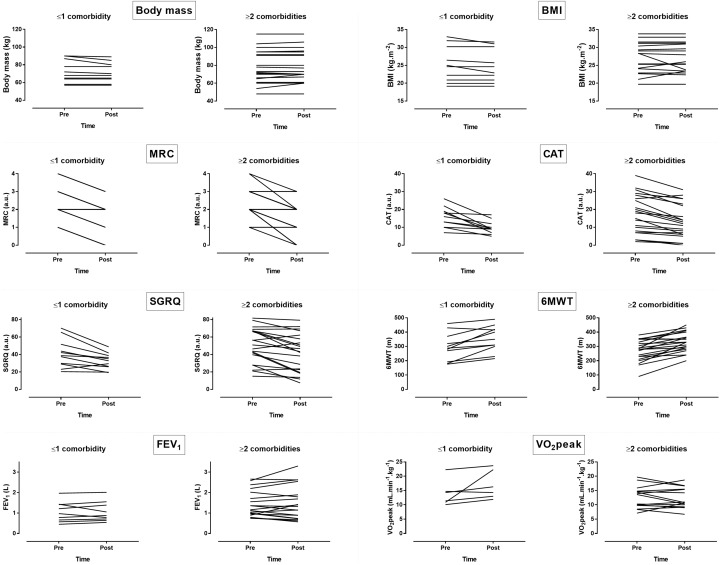
Inter-individual variability in changes of dyspnea, QOL and aerobic capacity following pulmonary rehabilitation. BMI = body mass index, FEV_1_ = forced expiratory volume in 1 s, MRC = modified Medical Research Council scale, CAT = COPD assessment test, SGRQ = St. George respiratory questionnaire, GOLD = global strategy for the diagnosis, management, and prevention of COPD, 6MWT = 6min walking test, VO_2_peak = peak oxygen uptake.

No statistically significant differences in performing the program were observed between the two groups. A large main effect of time was observed in MRC (*p* < 0.001, η^2^= 0.665), CAT (*p* < 0.001, η^2^= 0.621), SGRQ (*p* < 0.001, η^2^= 0.457), which decreased, and in 6MWT (*p* < 0.001, η^2^= 0.608), which increased across time (Figure 2, 3). No main effect of time was shown in body mass (*p* = 0.313, η^2^= 0.035), BMI (*p* = 0.251, η^2^= 0.045), FEV_1_ (*p* = 0.626, η^2^= 0.009) and VO_2_peak (*p* = 0.074, η^2^= 0.159). A large time ^∗^ group interaction was found in body mass (*p* = 0.012, η^2^= 0.198) with decrease in group 1 compared to group 2 and VO_2_peak (*p* = 0.025, η^2^= 0.238) with increase in group 1 compared to group 2. No time ^∗^ group interaction was observed in BMI (*p* = 0.249, η^2^= 0.046), MRC (*p* = 0.859, η^2^= 0.001), CAT (*p* = 0.279, η^2^= 0.039), SGRQ (*p* = 0.983, η^2^< 0.001), 6MWT (*p* = 0.888, η^2^= 0.001) and FEV_1_ (*p* = 0.783, η^2^= 0.003). In summary, the indices of dyspnea (MRC and CAT), QOL (SGRQ) and aerobic capacity (6MWT) improved across time. The combined effect of group and time was shown only in body mass and VO_2_peak.

The severity of the disease was not significantly associated with any of the observed changes after the rehabilitation program (rho ≤ 0.23, *p* ≥ 0.205). Only the reduction of dyspnea tended to be less – but not statistically significant – in severe and very severe COPD patients when compared to mild and moderate COPD patients (DMRC: 33% vs. 50%).

VO_2_peak showed a significant percentage increase of 25.7% in the subgroup of patients with none or one comorbidity (Group 1) compared to the subgroup of patients with two or more comorbidities (Group 2). However, the total number or/and specific comorbidities (CVD and diabetes mellitus) were not related to the general outcomes of pulmonary rehabilitation program, according to the correlation analysis. No harm was observed in participants.

## Discussion

Our prospective study demonstrated that all COPD patients improved, after a 13 week pulmonary rehabilitation program, in terms of dyspnea (MRC and CAT), QOL (SGRQ) and exercise tolerance in 6MWT, independently of the presence, the number or the nature of their comorbidities. We did not observe significant responses in FEV_1_ or VO_2_peak. To the best of our knowledge, this is the first study that evaluates the results of a rehabilitation program by both 6MWT and CPET.

It is well documented that pulmonary rehabilitation reduces dyspnea, improves QOL and increases exercise capacity in COPD patients (Spruit et al., 2013), especially in those who remain symptomatic despite maximal pharmacological therapy. Although COPD is characterized by multi – morbidity, which leads to worse disease symptoms, increased health care utilization and significant mortality, available data about the effect of comorbidities on pulmonary rehabilitation outcomes in COPD still conflicting (Crisafulli et al., 2008, 2010; Franssen and Rochester, 2014).

Most previous studies, that reported worse response after rehabilitation, in COPD patients with co-existing cardiovascular or metabolic diseases, were retrospective (Crisafulli et al., 2008; Vagaggini et al., 2009; Carreiro et al., 2013). In some studies, health status and/or 6MWT were negatively influenced by the presence of cardiac or metabolic diseases (Crisafulli et al., 2008; Vagaggini et al., 2009; Carreiro et al., 2013). On the contrary, another retrospective study, reported better response in 6MWT in COPD patients with metabolic diseases (Walsh et al., 2013). BMI > 25 kg m^-2^ was an independent predictor of improvement in 6MWT, but not in SGRQ, in one study (Vagaggini et al., 2009), whereas other studies did not observe any difference between obese and non-obese patients (Ramachandran et al., 2008; Greening et al., 2012). Psychological symptoms led to reduced improvement in dyspnea (Carreiro et al., 2013) and increased risk of drop-out of the program (Garrod et al., 2006).

Our study was prospective and our data were in partial accordance with previous studies. All COPD patients, independently of the number or the nature of comorbidities, achieved less dyspnea and better QOL after the rehabilitation program. The level of dyspnea was higher and tended to ameliorate less – but not statistically significant – in severe and very severe COPD patients, compared to moderate and mild patients. Probably more severe patients have more dyspnea and a significant degree remains even after the intervention. Cardiovascular or metabolic comorbidities were not associated with the outcomes of the rehabilitation program.

The objective performance of participants was measured by both 6MWT and CPET. All patients improved in 6MWT, but not in workload or VO_2_peak. In previous studies the most commonly assessed test was the 6MWT. It is reliable, simple, and of low cost. Furthermore, 6MWT resembles daily activities, as patients use to walk and not to cycle. However, changes in the 6MWT are not significantly correlated to changes in other indices of exercise performance (Ong et al., 2004), as it only measures a narrow range of the physical performance spectrum. Also, the 6MWT appears to be less sensitive to intervention than the CPET (Fotheringham et al., 2015). Group 1 achieved significantly better VO_2_peak compared to Group 2, after the rehabilitation program. This was in accordance with a previous study which showed that the improvement in exercise capacity was significantly greater in patients without significant comorbidities (Crisafulli et al., 2008).Probably patients with better baseline condition have greater possibility for improvement.

The amelioration of symptoms and exercise capacity, as expressed by 6MWT, in the whole population – independently of coexisting comorbidities – can be explained by the fact that physical activity and regular exercise are beneficial not only for patients with COPD, but also for patients with CVD, musculoskeletal disease, obesity, diabetes mellitus and most other chronic medical conditions (Franssen and Rochester, 2014). Moreover, in patients with heart failure or diabetes mellitus type 2, prescribed exercise, involving aerobic and resistance training modalities, is also evidence-based treatment (McMurray et al., 2012). Thus, pulmonary rehabilitation is beneficial for individuals with COPD and comorbidities, as it has positive effects on both.

The major limitation of our study is the small number of the patients. Besides, the severity of their comorbidities is not quantified and we considered only the number of presented comorbidities (i.e., none or one versus two to nine). Also, a major point of discussion is whether the results suggest real pathophysiologic differences in the response to pulmonary rehabilitation or are due to a greater deconditioning in chronic illness patients. On the other hand, strength of the study was its novelty as limited information existed so far about the role of the number of comorbidities on the effectiveness of a rehabilitation program for COPD (Negewo et al., 2015). Considering the high prevalence of COPD (Sobrino et al., 2017) and the key role of comorbidities (Koskela et al., 2014), the findings of the present study were of great practical importance for practitioners working with COPD.

## Conclusion

We conclude that all COPD patients benefit from a pulmonary rehabilitation program, independently of comorbidities. More severe patients and patients with ≥2 comorbidities have inferior outcomes when compared to moderate or to patients with 0–1 comorbidity. More studies are required to evaluate the effects of this intervention in the natural history of COPD and comorbidities.

## Data Availability

The raw data supporting the conclusions of this manuscript will be made available by the authors, without undue reservation, to any qualified researcher.

## Ethics Statement

All procedures performed in studies involving human participants were in accordance with the ethical standards of the Ethics Committee of the National and Kapodistrian University of Athens, School of Medicine at Alexandra Hospital of Athens and with the 1964 Helsinki declaration and its later amendments or comparable ethical standards. The Ethics Committee of the National and Kapodistrian University of Athens, School of Medicine at Alexandra Hospital of Athens, approved the study. All participants provided written informed consent.

## Author Contributions

MC, AR, and GT acquired and analyzed the data. PN, BK, and TR interpreted the data for the work. All authors made substantial contributions to the conception or design of the work, drafted the work or revised it critically for important intellectual content, provided final approval of the version to be published, and agreed to be accountable for all aspects of the work in ensuring that questions related to the accuracy or integrity of any part of the work are appropriately investigated and resolved.

## Conflict of Interest Statement

The authors declare that the research was conducted in the absence of any commercial or financial relationships that could be construed as a potential conflict of interest.

## Supplementary Material

DATA SHEET S1 Pre-intervention values for each participant.

DATA SHEET S2 Post-intervention values for each participant.

## Abbreviations

6MWT: six minute walking test
ATS: American Thoracic Society
BMI: body mass index
CAT: COPD assessment test
COPD: chronic obstructive pulmonary disease
CPR: cardiopulmonary resuscitation
CVD: cardiovascular disease
GOLD: global initiative for obstructive lung disease
MRC: medical research council
QOL: quality of life
SGRQ: St. George respiratory questionnaire
VO_2_peak: Peak oxygen uptake
VTE: systemic venous thromboembolism
