# Supplementary material for: Subjective and Objective Outcomes in Patients With COPD After Pulmonary Rehabilitation – The Impact of Comorbidities
Source: Front Physiol. 2019 Mar 22;10:286. doi: 10.3389/fphys.2019.00286 (PMC6438881; doi:10.3389/fphys.2019.00286)
Supplement: DATA SHEET S1 — Pre-intervention values for each participant. [file Data_Sheet_1.PDF]

	MRCpre	CATpre	SGRQpre	FEV1pre (lt	FEV1%pre	FVCpre (lt)	FVC%pre	FEV1/FVC
1	2	13	43,86	1,43	42,54	3,45	80,31	41,46
2	4	18	51,69	0,57	30	1,26	54	49,96
3	4	21	67,01	0,98	39	2,6	88	39
4	4	26	70,18	0,66	22,49	1,68	45,09	39,06
5	1	3	27,93	1,54	46,36	2,72	64,6	56,72
6	3	39	81,63	1,36	48,07	2,32	63,34	58,48
7	1	3	21,05	2,02	72,37	2,96	81,5	68,21
8	3	16	38,61	0,78	32	1,82	59	42,82
9	3	25	67,17	0,72	28,97	1,33	45,38	54,16
10	3	18	65,46	0,45	16	1,22	37	37
11	1	22	29,06	1,41	49	2,45	67	58
12	3	10	42,16	0,97	26	2,22	47	44
13	2	20	41,97	1,04	48	1,56	56	59
14	2	2	15,08	2,38	62	3,32	67	70
15	2	7	43,04	1,71	61	3,74	67	68
16	2	7	39,72	2,2	78	3,74	101	59
17	3	29	67,24	0,79	31,09	2,06	62,07	38,15
18	3	28	66,28	1,16	43,23	1,68	47,51	68,97
19	3	19	56,16	1,36	44,52	2,39	61,31	56,75
20	2	19	23	0,68	22,68	1,51	40,11	44,82
21	2	7	37,46	1,97	58,89	3,42	80	57,46
22	3	15	46,24	0,74	29,06	1,48	44,18	50,27
23	3	31	71,58	0,95	28,8	2,26	52,6	42,22
24	1	8	21,95	2,58	67	3,7	82	70
25	3	14	56,54	0,75	37,9	1,64	69,2	45,72
26	2	10	29,79	1,89	54,2	4,29	99	44
27	2	18	50,88	2,66	69,54	4,53	92,29	58,78
28	3	32	79,19	1	43,8	2,07	76,1	48,21
29	2	11	27,83	1,55	49,8	3,32	81,6	46,57
30	1	13	20,31	1,21	39,76	2,94	75,86	41,1
31	2	10	43,77	0,89	30,76	1,71	45,31	51,93
32	4	26	68,8	1,12	49,2	2,55	94,4	43,67

TLC%pre	FRC%pre	RV%pre	TLCO%pre	6MWDpre(	V02maxpre (L/Kg/min)
132,9	222	256,6	25,7	270	14,7
288	447	128		176	11,4
100	128	115	37	180	18,67
114,2	191,8	237,2	16,47	180	11,1
84,87	100,53	137,64	65,92	350	6,3
74,49	75,58	94,8	58,49	355	14,9
72,5	59,8	77,8	67,9	380	14,09
115	175	217	21	320	10,08
130,8	214,6	282,4	17,8	230	
34	42	48	4	192	
				280	22,29
125	211	307	40	288	
60	52	66		350	19,72
82	91	115	62	280	14,91
71	66	85	62	210	14,8
84	80	82	86	353	14,6
53,4	NO	63,7	37,4	305	10,3
60,1	97,6	86	28,57	280	13,6
113,7	174,2	204,5	25,64	90	8,4
53,5	59,9	74,1	32,9	370	
104,1	134,9	158	51,7	300	14,3
125,3	191,1	237,1	33,1	320	10
			36,2	170	8,5
				285	14,4
79,1	83,6	106,7	26,6	270	9,8
88	95,4	11,8	37,2	430	20,9
93,8	96,2	131,2	84,3	290	13,9
69,8	90,8	87	26,4	200	7,1
79,4	77,5	87,9	35,3	330	15,9
154,2	224,8	310	44,91	460	10,9
59,1		80	3,18	230	
109		155,3		240	10
