# Supplementary material for: Subjective and Objective Outcomes in Patients With COPD After Pulmonary Rehabilitation – The Impact of Comorbidities
Source: Front Physiol. 2019 Mar 22;10:286. doi: 10.3389/fphys.2019.00286 (PMC6438881; doi:10.3389/fphys.2019.00286)
Supplement: DATA SHEET S2 — Post-intervention values for each participant. [file Data_Sheet_2.PDF]

	MRCpost	CATpost	SGRQpost	FEV1post (l	FEV1%post	FVCpost (lt	FVC%post	FEV1/FVC
1	2	9	32,7	1,05	31,13	2,55	59,22	41,15
2	3	9	38,71	0,64	35,39	1,33	60	48,68
3	2	14	57,79	0,72	27	1,71	53	56,29
4	3	15	48,68	0,71	24	1,6	42,82	44,36
5	0	0	7,38					
6	2	31	79,26	1,32	47,17	2,49	68,58	52,83
7	1	1	11,77	1,79	64,19	2,31	63,52	77,63
8	2	12	36,63	0,88	36	1,49	49	48,03
9	2	14	42,23					
10	2	17	41,61	0,54	18,3	1,48	38	35,96
11	0	9	19,14	1,55	62	2,67	85	57,8
12	2	9	35,07	0,78	21,8	1,72	37,2	32,13
13	0	13	18,3	1,15	54	1,3	48	78
14	0	1	13,67	2,63	69	3,53	71,8	71
15	1	7	19,32	1,89	53	2,48	55	76,25
16	2	5	28,65	2,55	91	3,39	95	75,35
17	2	26	50,76	0,57	22	1,57	47	36,6
18	3	23	49,09	1,4	52,78	1,89	54,03	73,78
19	3	12	62,21	1,14	37,31	2,29	58,72	49,65
20	2	7	29,44					
21	1	6	26,33	2,01	61,1	3,63	86,2	55,32
22	2	6	53,34	0,68	26,71	1,32	39,75	51,17
23	2	22	71,84	0,89	26,8	1,84	41,3	51,18
24	1	6	23,49	3,3	98,42	3,95	91,05	83
25	2	11	43,25	0,62	32,18	1,4	60,21	44,52
26	1	5	25,87					
27	1	16	46,65	2,62	68,5	4,3	87,7	60,93
28	2	26	67,17	0,73	32,66	1,32	49,44	55,23
29	1	9	22,51	1,69	55,09	3,14	78,22	53,79
30	0	10	19,69	1,39	46,16	2,83	73,67	48,99
31	2	8	38,23	1,42	49	2,95	78	
32	3	28	69,24	0,89	39,5	2,14	79,19	41,57

TLC%post	FRC%post	RV%post	TLCO%post	6MWDpost	V02maxpost (L/Kg/min)
138,4	247,2		20,26	310	14,3
159,1	233,6		8,75	215	13
100	128		37	330	16,6
107,5	180,2		11,9	300	
				415	
138	197,7			350	
71	62,6		60,2	430	
76	87		29	350	11,9
				280	
				230	
112	149	178	104	420	23,7
				310	
				350	16,7
				310	
72	91	104	51	290	15,5
88	84	99	75	400	14,2
100,21	132,18	175,27	65,84	365	10
51,8	92,2	60,9	31,64	290	10,3
169, 2	267,2	371,5	22,83	200	6,7
				450	
94,7	120,4	133,1	49,3	400	16,3
59,1	71,6	93,2	14	330	9
114,8	179,1	251,5	33,8	240	9,2
112,2	151,6	170,6	91,69	450	10,8
66,9	80,4	98,7	13,5	360	9,3
				415	
89,66	81,26	118,53	74,79	330	15,4
72,6	97,1	117,2	16,1	270	10,7
76,72	84,85	89,35	34,12	410	18,7
78,89	72,66	99,72	48,69	490	22,3
				240	6,9
76		102	21,4	300	11
